# Homotypic Cancer Cell Membranes Camouflaged Nanoparticles for Targeting Drug Delivery and Enhanced Chemo-Photothermal Therapy of Glioma

**DOI:** 10.3390/ph15020157

**Published:** 2022-01-27

**Authors:** Yajing Ren, Chenlin Miao, Liang Tang, Yuxiang Liu, Pinyue Ni, Yan Gong, Hui Li, Fuxue Chen, Shini Feng

**Affiliations:** 1 School of Life Sciences, Shanghai University, No.381, Nanchen Road, Shanghai 200444, China; yajingren0415@163.com (Y.R.); miaochenlin1002@163.com (C.M.); liuyuxiang1998@126.com (Y.L.); qq310304204@126.com (P.N.); yangong0122@163.com (Y.G.); huili1870805069@126.com (H.L.); 2 School of Environmental and Chemical Engineering, Shanghai University, No.381, Nanchen Road, Shanghai 200444, China; tang1liang@shu.edu.cn

**Keywords:** graphene quantum dot, targeted drug delivery, photothermal therapy, chemo-therapy

## Abstract

Glioma is among the deadliest types of brain cancer, for which there currently is no effective treatment. Chemotherapy is mainstay in the treatment of glioma. However, drug tolerance, non-targeting, and poor blood–brain barrier penetrance severely inhibits the efficacy of chemotherapeutics. An improved treatment method is thus urgently needed. Herein, a multifunctional biomimetic nanoplatform was developed by encapsulating graphene quantum dots (GQDs) and doxorubicin (DOX) inside a homotypic cancer cell membrane (CCM) for targeted chemo-photothermal therapy of glioma. The GQDs with stable fluorescence and a superior light-to-heat conversion property were synthesized as photothermal therapeutic agents and co-encapsulated with DOX in CCM. The as-prepared nanoplatform exhibited a high DOX loading efficiency. The cell membrane coating protected drugs from leakage. Upon an external laser stimuli, the membrane could be destroyed, resulting in rapid DOX release. By taking advantage of the homologous targeting of the cancer cell membrane, the GQDs/DOX@CCM were found to actively target tumor cells, resulting in significantly enhanced cellular uptake. Moreover, a superior suppression efficiency of GQDs/DOX@CCM to cancer cells through chemo-photothermal treatment was also observed. The results suggest that this biomimetic nanoplatform holds potential for efficient targeting of drug delivery and synergistic chemo-photothermal therapy of glioma.

## 1. Introduction

Glioma is a common primary tumor of the central nervous system [1], accounting for about 45% of intracranial tumors. Glioma has a fast growth rate and a strong invasive ability, and it could infiltrate to the healthy brain tissues by proliferation and invasion, which makes it the most challenging intracranial tumor. Conventional therapy for glioma usually includes surgery, radiotherapy, and chemotherapy [2]. However, the median survival time of glioma patients has so far been only 12 months [3,4]. The current treatment of glioma faces several main obstacles, such as relapse, radiation tolerance, drug tolerance, and the blood–brain barrier [5]. Glioma poses a serious threat to human health and safety. Therefore, it is pivotal to further explore new treatment methods for glioma.

With the development of precision medicine, the nano-sized drug delivery system (NDDS) has become a research hotspot in the treatment of tumors. NDDS can accumulate in tumors via the enhanced permeability and retention (EPR) effects to achieve precise drug release [6,7,8]. However, the passive aggregation effect of nanoparticles is still limited, depending on the varying degrees of tumor vascularization and the permeability associated with tumor types and development stages [9,10,11]. To exert pharmacological activities, nano-therapeutics must overcome a cascade of physiological challenges, including blood circulation, cell toxicity, immune evasion, cell internalization, tumor targeting, etc. [12,13,14,15]. In recent years, graphene quantum dots (GQDs) nanomaterials have aroused great interest in the scientific community due to their unique physicochemical properties. As a zero micro-nano material, GQDs share some desirable properties of graphene oxide, including water solubility, stable fluorescence, and a variety of attributes, such as size, biocompatibility, excellent photothermal capacity, and the ability to cross the blood–brain barrier [16,17]. These properties make GQDs an excellent drug carrier and could be used in emerging tumor treatments, such as photothermal therapy (PTT) and biological imaging.

Inspired by biological systems in nature, cell membranes are increasingly being used to camouflage nanoparticles, which endow them with properties of natural cells, such as high biocompatibility, low immunogenicity, long circulation, and active targeting [18,19,20]. The camouflage of the red blood cell membrane for nanoparticles can prolong their circulation time in physical environments and help to escape the phagocytosis of the reticulo-endothelial system so that drugs could reach tumor site at higher concentrations [21,22,23,24]. Benefiting from the homing properties of stem cells [25,26], the mesenchymal stem cell membrane was wrapped on nanoparticles to bestow them with a targeting ability, enabling NDDS to escape from phagocytosis by the immune system to play a role upon reaching the tumor site [27,28,29]. It was reported that the tumor cell could aggregate to form metastatic cells, which was highly dependent on multimolecular-mediated cell–surface interactions, including tumor-specific binding proteins, such as the Thomsen–Friedenreich antigen and E-cadherin presenting on the membrane of the cancer cells [30,31]. Given the ability of homologous adhesion between tumor cells, it was speculated that the cancer cell membrane (CCM) coating could be applied to target the source cancer cells for anti-cancer drug delivery [32,33,34].

**Scheme 1:**
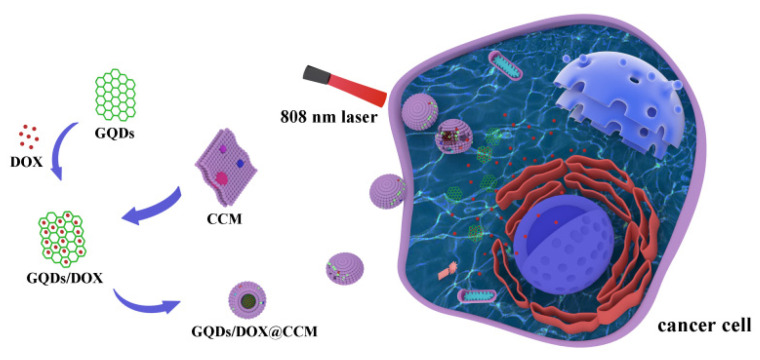
Schematic illustrating the preparation of GQDs/DOX@CCM for tumor-targeted chemo-photothermal combination therapy of glioma.

In this study, a novel cancer cell membrane camouflaged NDDS for targeted tumor treatment was developed (Scheme 1). The glioma cell membrane was utilized to camouflage GQDs with anti-cancer drug doxorubicin (DOX). The fabricated GQDs/DOX@CCM were fully characterized. Afterwards, cellular uptake and tumor targeting ability to homotypic glioma cells were investigated. Finally, the chemo-photothermal combination anti-cancer effect of GQDs/DOX@CCM was studied.

## 2. Results and Discussion

### 2.1. Characterization of GQDs

**Figure 1:**
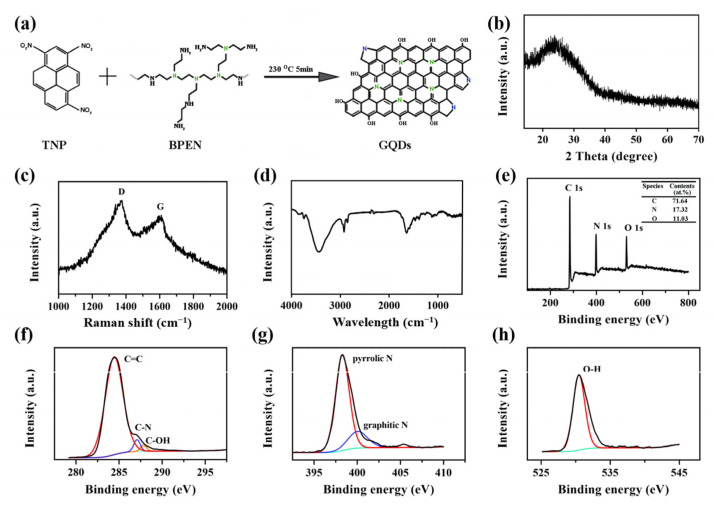
Characterization of GQDs. (**a**) Schematic diagram of the GQDs synthesis pathway. (**b**) XRD pattern of GQDs. (**c**) Raman spectrum of GQDs. (**d**) FTIR spectra of GQDs. (**e**) Survey XPS spectrum. (**f**) High-resolution C1s spectrum. (**g**) High-resolution N1s spectrum. (**h**) High-resolution O1s spectrum.

GQDs were synthesized according to the previous report [35]. As illustrated in Figure 1a, 1,3,6-trinitropyrene (TNP) and BPEI were dispersed in deionized water for ultrasonic treatment, and then they were reacted at 230 °C for 5 min. Finally, GQDs were obtained after a series of purification and dialysis. After GQDs were prepared, the morphology and structure were characterized. As shown in Figure 1b, GQDs have a broad peak at 23.9°, which corresponds to a wider layer (002) spacing of the graphite structure (3.71 Å), indicating the crystallinity of GQDs. The Raman spectrum shown in Figure 1c suggested that the ordered G band at 1617 cm^−1^ was stronger than the disordered D band at 1376 cm^−1^, with a large G to D intensity ratio of 1.2, which was consistent with a previous report [35]. The FTIR spectrum in Figure 1d showed a strong vibration at 1646 cm^−1^, ascribed to the C=C bonds, and a strong, rather broad vibration at 3471 cm^−1^ ascribed to the O-H bonds. The O-H signal was mainly ascribed to the hydroxyl functionalization of the GQDs, which was further confirmed by the XPS. As shown in Figure 1e, GQDs contained three obvious characteristics: C1s at 284.85 eV, N1s at 398.84 eV, and O1s at 531.06 eV, respectively. The C1s spectrum (Figure 1f) exhibited three characteristic peaks corresponding to C=C (284.43 eV), C-N (287.08 eV), and C-OH (287.83 eV). The N 1s spectrum (Figure 1g) showed the binding energy at 398.28 eV and 400.05 eV, corresponding to pyrrolic N and graphitic N, respectively. This result revealed that the fabricated GQDs were nitrogen-doped quantum dots. Besides this, the oxygen-containing species referred to hydroxyl (-OH) (Figure 1h), as confirmed by the C1s and O1s spectra, which further accounted for the good water solubility of GQDs.

**Figure 2:**
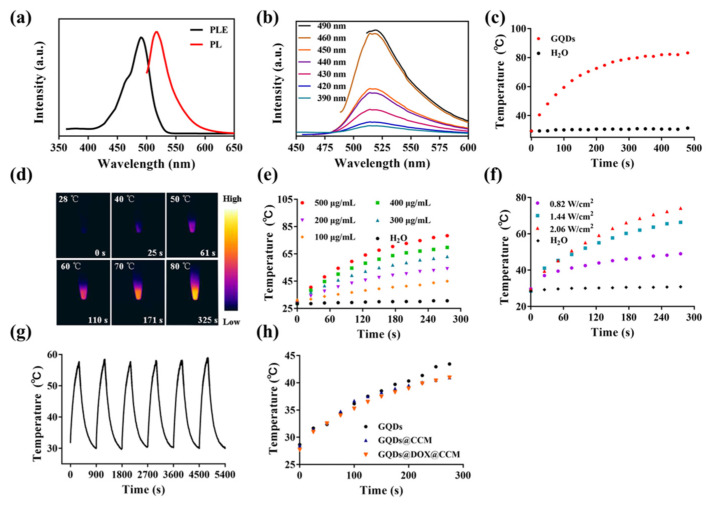
Characterization of GQDs. (**a**) PL and excitation (PLE) spectra of GQDs. (**b**) PL spectra of GQDs excited at different wavelengths varied from 390 to 490 nm. (**c**,**d**) The temperature change of the GQDs solution (500 μg/mL) under NIR laser irradiation (808 nm, 1.44 W/cm^2^) for 8 min. (**e**) The temperature change of the GQDs solution under NIR laser irradiation (808 nm, 1.44 W/cm^2^) with different concentrations for 5 min. (**f**) The temperature change of the GQDs solution of 300 μg/mL under 808 nm laser irradiation with different power for 5 min. (**g**) The photostability of GQDs of 200 μg/mL under NIR laser irradiation (808 nm, 1.44 W/cm^2^). (**h**) The temperature change of GQDs, GQDs@CCM, and GQDs/DOX@CCM solutions under NIR laser irradiation (808 nm, 1.44 W/cm^2^), and the GQDs concentration in all groups was 100 μg/mL.

As a novel fluorescent probe, the photoluminescence properties of GQDs were investigated. As shown in Figure 2a, the maximum excitation and emission wavelength of GQDs were 490 nm and 520 nm, respectively. The emission peak of GQDs hardly moved, while the excitation wavelength changed from 390 to 490 nm (Figure 2b). The distinguished luminous performance endowed GQDs with a potential application in tumor tracing and diagnosis. Thereafter, the photothermal performance of GQDs was verified. The GQDs solution with a concentration of 500 μg/mL was exposed to the near-infrared (NIR) laser (808 nm, 1.44 W/cm^2^), and the photothermal curves were recorded. As shown in Figure 2c, the temperature of the GQDs solution rose to 84 °C after 8 min of laser irradiation, while the aqueous solution did not change significantly. This result was further verified and visualized in Figure 2d. Results in Figure 2e,f suggested that the photothermal performance of GQDs exhibited concentration and laser power-dependent manner. After six cycles of laser irradiation, the photothermal conversion capability of GQDs remained stable (Figure 2g). After the photothermal sensitivity of GQDs was characterized, we further explored the photothermal conversion efficiency of GQDs. It was seen from Figure S1a that GQDs have a clear absorption peak in the near-infrared region, which was due to the doping of nitrogen and the presence of oxygen-containing functional groups. The absorbance of GQDs at 808 nm was 0.160. Then, the GQDs solution was exposed to a NIR laser (808 nm, 1.44 W/cm^2^) for 300 s and cooled naturally. The temperature change in Figure S1b showed that under the irradiation of the 808 nm laser, the temperature of the GQDs solution rose from 25 to 66 °C, and after 600 s of natural cooling, it dropped to 30 °C. The time constant (τs) of GQDs was calculated to 204.66 s (Figure S1c), and the photothermal conversion efficiency of GQDs was further calculated to be 49.97%. After cell membrane encapsulation, the photothermal ability of GQDs@CCM and GQDs/DOX@CCM showed only slight changes compared with GQDs (Figure 2h). These results demonstrated the excellent photothermal sensitivity of GQDs and their promising potential for application in the photothermal therapy of tumors.

### 2.2. Preparation and Characterization of GQDs/DOX@CCM

The biomimetic drug delivery nanoplatform GQDs/DOX@CCM was synthesized as follows: GQDs were mixed with the anticancer drug hydrochloride doxorubicin (DOX) for π-π stacking and electrostatic interaction at room temperature for 24 h. Then, the mixture was co-extruded with CCM, which was gained from freshly harvested BV2 cells. After ultracentrifugation, the GQDs/DOX@CCM was obtained and characterized. 

**Figure 3:**
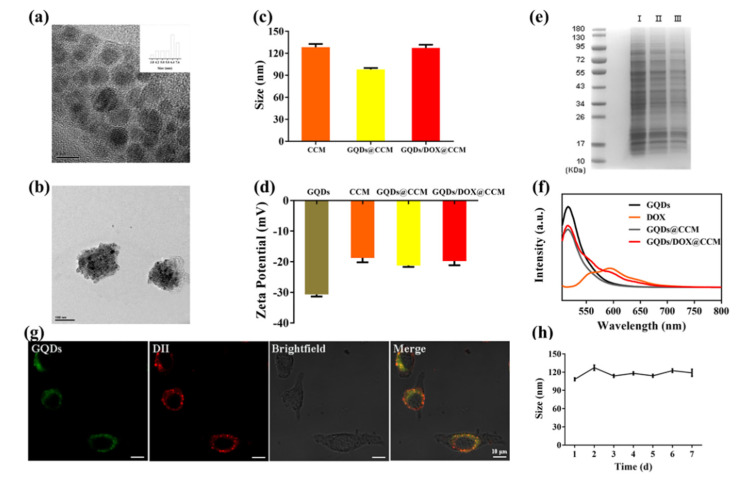
Characterization of GQDs/DOX@CCM. (**a**) TEM image of GQDs. Scale bar = 5 nm. (**b**) TEM image of GQDs/DOX@CCM. Scale bar = 100 nm. (**c**) DLS analysis of CCM, GQDs@CCM, and GQDs/DOX@CCM. (**d**) Zeta potentials of GQDs, CCM, GQDs@CCM, and GQDs/DOX@CCM. (**e**) SDS-PAGE protein analysis of I CCM, II GQDs@CCM, and III GQDs/DOX@CCM. (**f**) PL spectra of GQDs, DOX, GQDs@CCM, and GQDs/DOX@CCM. (**g**) CLSM images of the GQDs@CCM illustrating the colocalization of the GQDs and CCM (DII channel). Scale bar = 10 μm. (**h**) Stability of GQDs/DOX@CCM in PBS (pH = 7.4) by measuring the particle size.

The morphology and structure of GQDs and GQDs/DOX@CCM were visualized by transmission electron microscopy (TEM). As a zero-dimensional nanomaterial, GQDs exhibited a clear lattice structure with an ultra-small particle size of 5.8 ± 2 nm (Figure 3a). After being encapsulated in CCM with DOX, GQDs/DOX@CCM showed an increased particle size of approximately 130 ± 10 nm, which was further confirmed by the hydrodynamic particle size measured by DLS (Figure 3b,c). As shown in Figure 3d, the surface charge of the CCM was determined to be −18.76 ± 1.41 mV, which was consistent with previous reports [36,37]. The GQDs/DOX@CCM exhibited a surface potential (−19.8 ± 1.34 mV) similar to that of CCM, suggesting a successful membrane coating. Subsequently, the protein contents of GQDs/DOX@CCM were assessed by SDS-PAGE. Figure 3e indicated that the compositions of membrane proteins were mostly retained on GQDs/DOX@CCM. 

To further verify the integrity of the core-shell particle structure, fluorescent dye DII was used to stain the CCM of GQDs@CCM. Figure 3f showed that the emission peak of GQDs was around 480 nm when excited at 405 nm, and the emission peak of DOX was around 600 nm. The characteristic peaks of GQDs and DOX were both shown in GQDs/DOX@CCM, which indicated the successful loading of GQDs and DOX. After being co-cultured with BV2 cells for 1 h, GQDs@CCM was visualized by CLSM. As shown in Figure 3g, the green fluorescent signals from the GQDs and red fluorescence from the membrane exhibited a high degree of colocalization, indicating the structural integrity of GQDs@CCM. Moreover, the long-term stability of GQDs/DOX@CCM was measured by DLS, which revealed that the GQDs/DOX@CCM was stable at around 33–36 °C within 7 d (Figure 3h). These results demonstrated the successful synthesis of GQDs/DOX@CCM with an intact natural surface membrane protein and long-term stability.

### 2.3. Biocompatibility In Vitro

**Figure 4:**
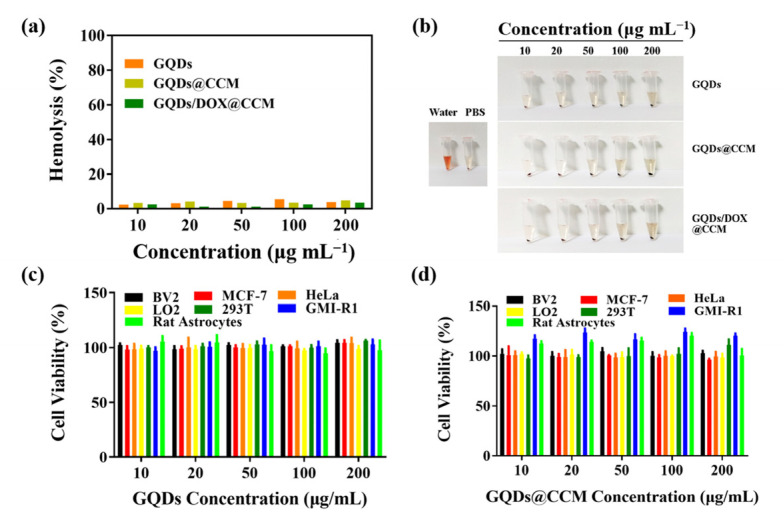
Biocompatibility in vitro. (**a**,**b**) Hemolysis quantification of red blood cells incubated with deionized water, PBS, GQDs, GQDs@CCM, and GQDs/DOX@CCM. (**c**,**d**) Cytotoxicity of GQDs and GQDs@CCM to various cell lines with different concentrations for 24 h.

Considering the importance of the biocompatibility of nanomaterials for clinical applications, we investigated the biosafety of GQDs and GQDs-based membrane camouflaged nanoparticles through blood compatibility and cell cytotoxicity. It was reported that incompatible materials can break the red blood cells, activate and aggregate platelets, and then induce the formation of thrombosis when entering the blood [38]. Therefore, a hemolysis assay is an important index to explore the biocompatibility of materials. The results of blood compatibility (Figure 4a,b) showed that the hemolysis rate was less than 5% after incubating with GQDs@CCM or GQDs/DOX@CCM of different concentrations for 4 h. To further verify the cytotoxicity of GQDs@CCM, cells from different sources were co-cultured with GQDs or GQDs@CCM for 24 h. The CCK-8 result (Figure 4c,d) showed that cell viability in all groups remained above 95%, even at a high concentration of 200 μg/mL. These results suggested the high biosafety and bio-compatibility of GQDs and GQDs@CCM.

### 2.4. DOX Encapsulation and Releasing

**Figure 5:**
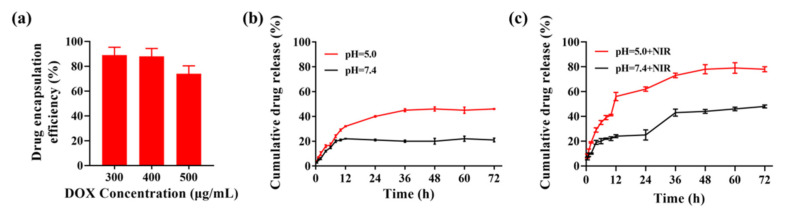
Drug loading capacity and release behavior of GQDs/DOX@CCM. (**a**) Encapsulation efficiency of DOX with initial concentrations of 300, 400, and 500 μg/mL. (**b**) DOX release from GQDs/DOX@CCM at pH 5.0 and pH 7.4. (**c**) DOX release from GQDs/DOX@CCM after 5 min of NIR laser irradiation (808 nm, 1.44 W/cm^2^).

Drug loading efficiency was investigated under the presence of DOX with different concentrations. It was shown that the DOX encapsulation efficiency was calculated to be 89% ± 6.37, 88% ± 6.19, and 74% ± 6.54 when the initial added DOX concentration was 300 μg/mL, 400 μg/mL, and 500 μg/mL, respectively (Figure 5a). Next, the drug release behavior of GQDs/DOX@CCM was detected at about 33–36 °C. As shown in Figure 5b, the drug release rate in a pH 5.0 solution was about 46% ± 0.4 at 72 h, which was significantly higher than that in pH 7.4 (22% ± 2.1), suggesting pH-dependent drug release behavior of GQDs/DOX@CCM. Moreover, the release rate of DOX from GQDs/DOX@CCM with NIR laser irradiation (808 nm, 1.44 W/cm^2^) was significantly improved compared with the non-irradiated group (48% ± 1.2 vs. 78% ± 2.0) (Figure 5c). The laser irradiation induced amhigh temperature, which helped boosting DOX release. These results suggest the favorable controlled release behavior of GQDs/DOX@CCM for reducing the side effects of DOX to normal tissues.

### 2.5. In Vitro Homologous Targeting

**Figure 6:**
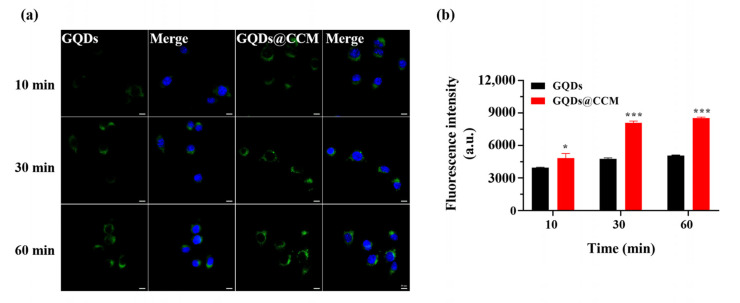
Cellular uptake of GQDs and GQDs@CCM. (**a**) CLSM images of BV2 cells incubated with GQDs or GQDs@CCM at a GQD concentration of 200 μg/mL for different time periods. Scale bar = 10 μm. (**b**) Quantitative analysis of BV2 cell uptake by FCM in different time periods. The differences among groups were determined by a one-way ANOVA analysis followed by the Tukey’s post-test: * *p* <0.05, *** *p* < 0.001.

When cultured with BV2 cells, GQDs tended to locate at the cytoplasm and exhibit stable green fluorescence (Figure 6a). The cellular internalization of GQDs and GQDs@CCM in BV2 cells were investigated by CLSM and FCM. As displayed in Figure 6a, GQDs could be internalized and located in the cytoplasm of cancer cells. However, after being wrapped by the BV2 cell membrane, a stronger green fluorescence was observed in BV2 cells at the same incubation time. This result was further verified by the quantitative analysis of FCM (Figure S2a–c). After 60 min of co-culturing, the fluorescence intensity of intracellular GQDs@CCM was 1.6 times that of GQDs at the same GQDs concentration of 200 μg/mL (Figure 6b). The enhanced cellular uptake of GQDs@CCM could be attributed to the surface adhesion molecules in the cell membrane, which endowed the nanocomposites with the homotypic tumor self-recognition ability.

**Figure 7:**
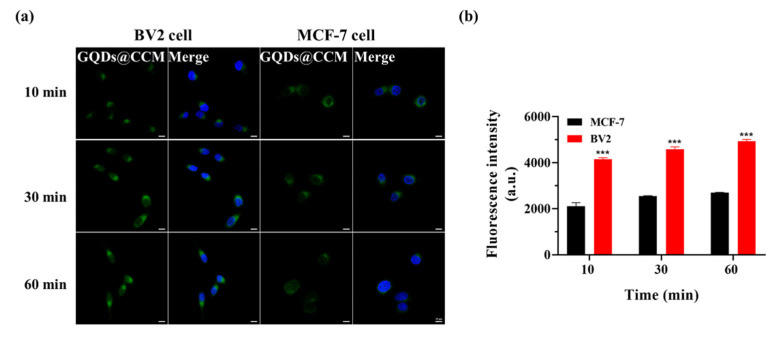
Homologous targeting of GQDs@CCM. (**a**) CLSM images of BV2 and MCF-7 cells incubated with GQDs@CCM at a GQDs concentration of 200 μg/mL for different time periods. Scale bar = 10 μm. (**b**) Quantitative analysis of cell uptake by FCM in different time periods. The differences among groups were determined by a one-way ANOVA analysis followed by the Tukey’s post-test: *** *p* < 0.001.

To further demonstrate the self-recognition ability of cancer cell membrane camouflaged GQDs with homotypic cancer cells, cellular internalization of BV2 cell membrane coated GQDs@CCM by BV2 cells and MCF-7 cells was studied. It was found that the fluorescence intensity originating from BV2 cell membrane coated NPs was far superior in the source BV2 cells than in heterotypic cells MCF-7 after co-culturing for 1 h, which approximated to 2.0–2.3-fold in terms of the mean fluorescence intensity inside cells (Figure 7a,b). These results were also validated by FCM (Figure S2d–f). Besides this, a more rapid cell internalization by the source cell membrane coated GQDs@CCM was found when the incubation period was further prolonged. These results proved the high specific self-recognition affinity of GQDs@CCM to the source tumor cells.

### 2.6. Antitumor Efficacy In Vitro

**Figure 8:**
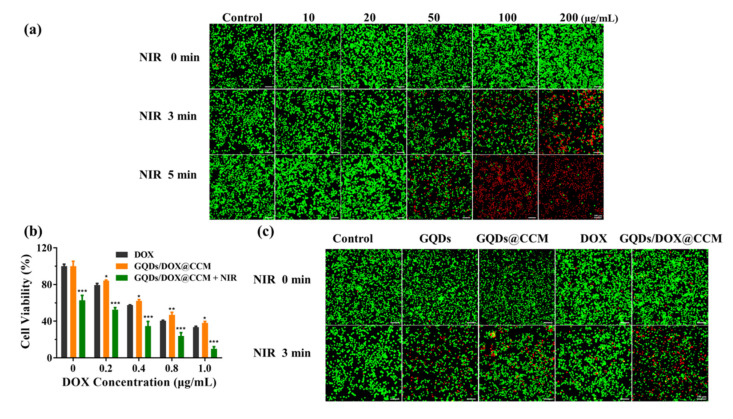
Antitumor efficacy in vitro. (**a**) The cytotoxicity of BV2 cells treated with different concentrations of GQDs for 4 h with NIR laser irradiation (808 nm, 1.44 W/cm^2^). Scale bar = 10 μm. (**b**) Relative BV2 cells viability after treatment with different concentrations of DOX, GQDs/DOX@CCM, and GQDs/DOX@CCM (NIR) for 24 h. (**c**) Calcein-AM/PI cell staining of BV2 cells subjected to different treatments. Scale bar = 10 μm. The differences among groups were determined by a one-way ANOVA analysis followed by the Tukey’s post-test: * *p* <0.05, ** *p* <0.01, *** *p* < 0.001.

Encouraged by the aforementioned results, we further investigated the anticancer effect of GQDs/DOX@CCM in vitro. Then GQDs were co-cultured with BV2 cells and subjected to different laser treatments. The cell survival situation was studied by using Calcein-AM and PI as probes to indicate the live (green) and dead (red) cells, respectively (Figure 8a). The result showed that GQDs treatment did not cause obvious cell death. However, after laser treatment, the red fluorescence of dead cells increased with the increase of the GQDs concentration and irradiation time, indicating their excellent photothermal performance in cancer treatment. Subsequently, the antitumor activity of GQDs/DOX@CCM by the combination of chemo-phototherapy in vitro was investigated. BV2 cells were treated with free DOX and GQDs/DOX@CCM for 24 h, and cell viability was evaluated through a CCK-8 assay (Figure 8b). The reduction in BV2 cell viability was dose-dependent in all groups. Both GQDs (under NIR) and free DOX could effectively inhibit cell proliferation. After being co-encapsulated in CCM, there was a slight reduction in the cytotoxicity of GQDs/DOX@CCM, which was mainly caused by the relatively long drug release time in cancer cells. However, after being irradiated by an external laser, less than 10% of cells survived. The significantly enhanced toxicity to cancer cells could be attributed to the cooperation of the homologous targeted delivery, superior photothermal effect, and controlled drug release responding to the temperature increment. These results were further confirmed by Calcein-AM/PI staining, which was consist with the viability assay (Figure 8c). The above results demonstrated that GQDs/DOX@CCM possess superior therapeutic performance in homogeneous cancer cells. However, further in vivo experiments are still needed before making a final statement on their antitumor effect.

## 3. Materials and Methods

### 3.1. Materials

Pyrene (purity > 98%) was obtained from TCI (Tokyo Chemical Industry, Tokyo, Japan). Polymers of branched polyethylenimine (BPEI) were obtained from Aladdin (Shanghai, China). A Cell Counting Kit-8 (CCK-8) and Calcein-AM/PI Double Staining Kit were purchased from dojindo (Dojindo Laboratories, Kumamoto, Japan). Dulbecco’s modified Eagle’s medium (DMEM), heat-inactivated fetal bovine serum (FBS), Doxorubicin (DOX), and trypsin were purchased from BI (Shanghai, China). Furthermore, 4,6-diamidino-2-phenylindole (DAPI) and DII were purchased from beyotime (Shanghai, China). 

### 3.2. Cell Culture

Mouse glioma cells (BV2), human cervical carcinoma cells (HeLa), human breast cancer cells (MCF-7), human normal liver cells (LO2), human embryonic kidney cells (293T), rat microglia (GMI-R1), and rat astrocytes cells were obtained from the Chinese academy of sciences. All cells were cultured in a DMEM medium supplemented with 1% penicillin–streptomycin and 10% fetal bovine serum at 37 °C in a humidified atmosphere of 90% humidity and containing 5% CO_2_.

### 3.3. Isolation of Cancer Cell Membrane

When BV2 cells grew to 90% confluence in culture flasks, cells were collected and suspended in a hypotonic cell lysis buffer, placed in 4 °C for 12 h, then disrupted by a D-130 homogenizer (Beijing, China) for 20 passes. The cell suspensions were centrifuged at 3000 rpm for 20 min at 4 °C; the resulting supernatant was collected and centrifuged again at 100,000× *g* for 30 min. The plasma membrane pellet was resuspended in PBS with PMSF and stored in −80 °C for further experiments.

### 3.4. Preparation of GQDs and GQDs/DOX@CCM

According to the previous report [39], the 1,3,6-trinitropyrene (TNP) was firstly prepared by the nitrification reaction of pyrene. Then, TNP and BPEI were dispersed in deionized water for ultrasonic treatment and placed in a dedicated reactor at 230 °C for 5 min. Finally, the pure GQDs were obtained after filtration and dialysis.

A total of 1 mL of DOX (800 μg/mL) was added to 1 mL of GQDs (400 μg/mL) at room temperature in the dark for 24 h. The mixture was co-extruded with 2 mL of BV2 cancer cell membrane vesicles through a 200 nm polycarbonate membrane for 20 passes. The unloaded DOX and GQDs were removed by centrifugation at 13,000× *g* for 10 min. 

### 3.5. Characterization

Transmission electron microscopy (TEM) images were carried out by a G2F20 microscope (Thermo Fisher Scientific, Wyman Street, Waltham, MA, USA). Raman spectra were recorded on a horiba evolution laser Raman spectrometer (Horiba Scientific, Piscataway, NJ, USA). X-ray photoelectron spectroscopy (XPS) measurements were performed using a K-Alpha^+^ X-ray photoelectron spectrometer (Thermo Fisher Scientific, Wyman Street, Waltham, MA, USA). X-ray diffraction (XRD) patterns were obtained with a D8 Advance X-ray diffractometer (Showa, Tokyo, Japan). The fluorescence spectrum was recorded with an LS55 fluorophotometer (PerkinElmer Inc., Waltham, MA, USA). UV-Vis-NIR spectroscopy was recorded on a U-4150 Ultraviolet visible near infrared spectrophotometer (Hitachi, Tokyo, Japan). The FTIR spectrum was recorded on the VERTEX70 Fourier infrared spectrometer (Bruker, Karlsruhe, Germany). Particle size and surface zeta potential were measured by dynamic light scattering (DLS) using Zetasizer equipment (Zetasizer Nano ZN, Malvern Analytical Ltd., Malvern, UK). The protein components were analyzed by sodium dodecyl sulfate-polyacrylamide gel electrophoresis (SDS-PAGE).

### 3.6. Photothermal Performance Measurements

GQDs with different concentrations were exposed to a near-infrared laser (808 nm) for 5 min with different power densities; the temperature of the solution was measured and recorded with an E53 infrared thermal imager (FLIR Systems Inc, Portland, OR, USA). In the photothermal stability experiment, the solution of GQDs was radiated with the near-infrared laser (808 nm, 1.44 W/cm^2^) for 5 min, and then the solution was cooled to room temperature naturally. After being repeated 6 times, the temperature was recorded, and the temperature curve was drawn. Different concentrations of GQDs were co-cultured with BV2 cells for 4 h and then irradiated by a near-infrared laser (808 nm, 1.44 W/cm^2^) for 3 or 5 min. Cell mortality was measured through the Calcein-AM/PI Double Staining Kit.

### 3.7. Photothermal Conversion Efficiency Measurement

The photothermal conversion efficiency of GQDs was investigated by irradiating the centrifuge tube containing a dispersion of GQDs at 300 μg/mL (808 nm, 1.44 W/cm^2^). The temperature of the GQDs solution was measured every 25 s by an infrared thermal imager (FLIR Systems Inc, Portland, OR, USA).

Q_Dis_ can be determined by the following Equation [40,41]:

(1) $$Q_{\mathrm{DIS}}=\frac{m_{D}c_{D}\Delta_{T}}{t}$$

where Q_Dis_ represents the baseline energy input of water, m_D_ is the mass of aqueous solution, c_D_ is the specific heat capacity of aqueous solution, Δ_T_ is the temperature change of aqueous solution during the 5 min laser irradiation (808 nm, 1.44 W/cm^2^), and t is the irradiation time (5 min).

h represents heat-transfer coefficient, A represents the surface area of the container, and hA can be determined by the following equation:

(2) $$\theta =\frac{T-T_{\mathrm{surr}}}{T_{\max}-T_{\mathrm{surr}}}$$

t = −τ_s_(lnθ) (3)

(4) $$\tau_{s}=\frac{m_{D}c_{D}}{\mathrm{hA}}$$

Here, θ is a dimensionless parameter, and τ_s_ is the time constant of the sample system. c_D_ and m_D_ represent the mass of GQDs (0.5 g) and heat capacity (4.2 J/g), T_max_ represents the maximum temperature of GQDs solution, and T_surr_ represents the ambient temperature. 

Photothermal conversion efficiency (η) can be determined by the following equation:

(5) $$\eta =\frac{\mathrm{hA}\left(T_{\max}-T_{\mathrm{surr}}\right)-Q_{\mathrm{DIS}}}{I\left(1-10^{-A\lambda }\right)}\times 100\%$$

I is the laser power, and Aλ is the absorption value at 808 nm.

### 3.8. Biocompatibility In Vitro

The blood of healthy Kunming mice was collected from the eyeball and then centrifuged several times to obtain red blood cells and suspended in PBS. A total of 0.2 mL of erythrocytes were mixed with I 0.2 mL PBS (negative control), II 0.2 mL of deionized water (positive control), and III 0.2 mL of GQDs, GQDs@CCM, or GQDs/DOX@CCM solutions of different concentrations. The mixtures were placed at room temperature (33–36 °C) for 4 h. Then, the supernatants were collected and measured at 540 nm to calculate the hemolysis rate after centrifugation at 10,000× *g* for 5 min.

(6) $$\mathrm{Hemolysis}\;\mathrm{rate}=\frac{A_{\mathrm{sample}}-A_{\mathrm{Negative}\;\mathrm{control}}}{A_{\mathrm{Positive}\;\mathrm{control}}-A_{\mathrm{Negative}\;\mathrm{control}}}\times 100\%$$

The cell viability was investigated by Cell Counting Kit (CCK)-8 assays. BV2, MCF-7, HeLa, LO2, 293T, GMI-R1, and rat astrocyte cells were seeded into 96-well plates (n = 5) at a density of 4000 cells per well. After adhering, the medium was taken out, and 100 μL of fresh medium containing GQDs or GQDs@CCM with various GQDs concentrations (10, 20, 50, 100, and 200 μg/mL) were added and co-cultured for another 24 h. Then, 10 µL of the CCK-8 solution was added to each well and incubated for 2 h. Finally, the absorbance at 450 nm was detected by a VICTOR X3 Multifunctional microplate reader (PerkinElmer Inc., Waltham, MA, USA).

### 3.9. Drug Encapsulation Efficiency and Drug Release In Vitro

To assess the drug loading capacity, GQDs/DOX@CCM was centrifuged to remove the unloaded DOX. The unloaded DOX was determined by measuring absorption at 480 nm using a Multifunctional microplate reader (Perkin Elmer, Waltham, MA, USA). The drug encapsulation efficiency was calculated using the following formula:

(7) $$\mathrm{Drug}\;\mathrm{encapsulation}\;\mathrm{efficiency}\;(\%)=\frac{\mathrm{Initial}\;\mathrm{mass}\;\mathrm{of}\;\mathrm{drug}-\mathrm{Mass}\;\mathrm{of}\;\mathrm{unload}\;\mathrm{drug}}{\mathrm{Initial}\;\mathrm{mass}\;\mathrm{of}\;\mathrm{drug}}\times 100\%$$

To measure the profile of drug release, 1.5 mL of GQDs/DOX@CCM (DOX concentration of 300 μg/mL) were dialyzed in 8 mL PBS with a different pH (7.4 or 5.0) at about 33–36 °C in the dark (MWCO = 3.5KD). At different time intervals, 500 μL of dialysis buffer was withdrawn to detect the amount of DOX released. After the measurement, the solution was poured back to maintain the constant total amount.

### 3.10. Cellular Uptake

BV2 cells were seeded into a 35 mm confocal dish (n = 3) at a density of 4000 cells per well and cultured for 24 h. Then, cells were cultured with GQDs or GQDs@CCM for 10 min to 60 min. Cells were washed three times and treated with paraformaldehyde for 20 min. After that, DAPI was added to label the nuclei for 5 min. Then, cells were washed with PBS and visualized by confocal laser scanning microscopy (CLSM) (Occult International Ltd., DE). For quantitative analysis, BV2 cells were seeded into 12-well plates (n = 3) at a density of 40,000 cells per well and cultured for 24 h; then, GQDs or GQDs@CCM were added with a final GQDs concentration of 200 μg/mL for 10 to 60 min; after the cells were washed and digested, the fluorescence of GQDs and GQDs@CCM were observed with a Flow cytometer (Beckman Coulter, Inc., S. Kraemer Boulevard Brea, CA, USA) at the PB450A channel.

### 3.11. Homologous Recognition In Vitro

BV2 cells and MCF-7 cells were seeded into a 35 mm confocal dish (n = 3) at a density of 4000 cells per well and cultured for 24 h. Then, cells were incubated with GQDs@CCM at a final GQDs concentration of 200 μg/mL from 10 to 60 min. After the cells were washed and fixed with paraformaldehyde for 20 min, the cells were stained with DAPI for 5 min. The internalization of GQDs or GQDs@CCM by cells was observed via CLSM. Simultaneously, flow cytometry (FCM) was also used to evaluate the specific targeting of cells. BV2 cells and MCF-7 cells were seeded into 12-well plates (n = 3) at a density of 40,000 cells per well and cultured for 24 h. Then, cells were incubated with GQDs@CCM from 10 to 60 min. After the cells were washed and digested, the fluorescence of BV2 and MCF-7 cells were observed with a Flow cytometer (Beckman Coulter, Inc., S.Kraemer Boulevard Brea, CA, USA) at the PB450A channel.

### 3.12. Anti-Tumor Effect In Vitro

BV2 cells were seeded into 96-well plates (n = 5) at a density of 4000 cells per well and cultured for 24 h. The cell culture medium was replaced with 100 μL of fresh medium containing an increased concentration of DOX and GQDs/DOX@CCM complexes, respectively. The treatment concentration of GQDs was 100 μg/mL in the GQDs/DOX@CCM group. After co-cultivation for 24 h, the cells were washed twice with PBS, and then they were incubated with 10 μL of CCK-8 solution for another 2 h. The absorbance at 450 nm was detected using a Multifunctional plate reader ((PerkinElmer Inc., Waltham, MA, USA). The PTT groups were exposed to an 808 nm laser with a power density of 1.44 W/cm^2^ for 3 min after co-cultivation for 4 h, with other conditions kept the same. The formula for calculating the cell viability (%) was listed as follows:

(8) $$\mathrm{Cell}\;\mathrm{viability}=\frac{A}{A_{0}}\times 100\%$$

Note: A is the OD_450_ of the treated BV2 cells; A_0_ is the OD_450_ of the untreated BV2 cells.

For the Calcein-AM/PI double-staining assay, BV2 cells were seeded in 35 mm confocal dishes (n = 3) at a density of 40,000 cells per well and co-cultivated with free DOX, GQDs, GQDs@CCM, and GQDs/DOX@CCM for 4 h. The concentration of GQDs was kept consistent at 100 μg/mL. Then, cells were washed three times with PBS and subjected to the light irradiation (808 nm, 1.44 W/cm^2^) for 3 min. After being washed by PBS, cells were stained with Calcein-AM and PI for 15 min at 37 °C. Finally, cells were visualized by CLSM.

### 3.13. Statistical Analysis

Data was reported as mean ± SD. The differences among groups were determined by a one-way ANOVA analysis followed by the Tukey’s post-test: (*) *p* < 0.05, (**) *p* < 0.01, (***) *p* < 0.001.

## 4. Conclusions

In summary, a cancer cell membrane biomimetic drug delivery system for chemo-photothermal combination therapy of homogeneous cancer cells was developed. The synthesized GQDs with favorable properties of excellent dispersibility, controllable size, stable fluorescence, and superior photothermal performance was utilized as a therapeutic agent and co-encapsulated with DOX in a BV2 cell membrane. The GQDs were not only capable of converting near-infrared light to heat for photothermal therapy, but they also improved the release of DOX for chemotherapy. Besides this, benefiting from the homologous targeting of the cancer cell membrane, the GQDs/DOX@CCM can actively target BV2 cells in vitro, resulting in a higher cellular uptake. The anti-tumor results demonstrated the superior killing efficiency of GQDs/DOX@CCM to cancer cells through chemo-photothermal treatment. Based on this study, the fabricated GQDs/DOX@CCM are capable of providing an effective combination strategy for precision oncology therapy.

## Data Availability

The data are contained within the article and Supplementary Materials.

## Supplementary Materials

The following supporting information can be downloaded at https://www.mdpi.com/article/10.3390/ph15020157/s1: Figure S1: characterization of GQDs. (a) UV-Vis-NIR spectrum of GQDs. (b) Photothermal effect of the GQDs solution (300 μg/mL) exposed to the NIR laser (808 nm, 1.44 W/cm^2^). The lasers were shut off after 300 s irradiation. (c) Plot of cooling time versus negative natural logarithm of the temperature driving force obtained from the cooling period after the NIR irradiation (808 nm, 1.44 W/cm^2^). Figure S2: Flow cytometry analysis. (a–c) Mean fluorescence intensity of BV2 cells after 10, 30, and 60 min incubation with GQDs or GQDs@CCM; the final concentration of GQDs was 200 μg/mL. (d–f) Mean fluorescence intensity of MCF-7 and BV2 cells after 10, 30, and 60 min incubation with GQDs@CCM; the final concentration of GQDs was 200 μg/mL. Figure S3. Homologous targeting of GQDs@CCM. (a) CLSM images of GMI-R1 and Rat astrocytes cells incubated with GQDs@CCM at GQDs concentration of 200 μg/mL for different time period. Scale bar = 10 μm. (b,c) Quantitative analysis of cell uptake by FCM in different cell lines.
