# Supplementary material for: Homotypic Cancer Cell Membranes Camouflaged Nanoparticles for Targeting Drug Delivery and Enhanced Chemo-Photothermal Therapy of Glioma
Source: Pharmaceuticals (Basel). 2022 Jan 27;15(2):157. doi: 10.3390/ph15020157 (PMC8879672; doi:10.3390/ph15020157)
Supplement: Supplementary file 1 [file pharmaceuticals-15-00157-s001.zip › pharmaceuticals-1521731-supplementary.pdf]

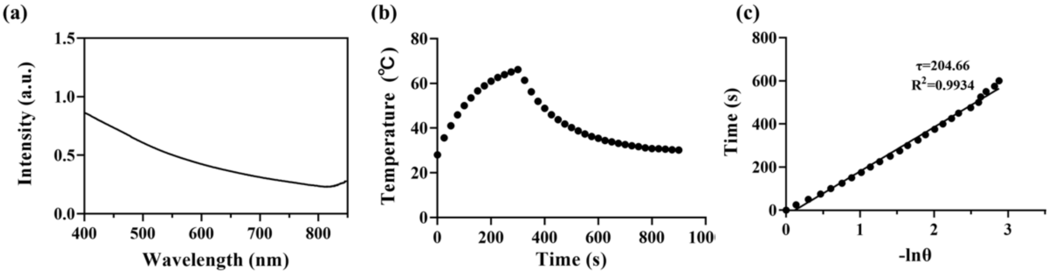

Figure S1. characterization of GQDs. (a) UV-Vis-NIR spectrum of GQDs. (b) Photothermal effect of GQDs solution (300 μg/mL) exposed to the NIR laser (808 nm, 1.44 W/cm^2). The lasers were shut off after 300 s irradiation. (c) Plot of cooling time versus negative natural logarithm of the temperature driving force obtained from the cooling period after the NIR irradiation (808 nm, 1.44 W/cm^2).

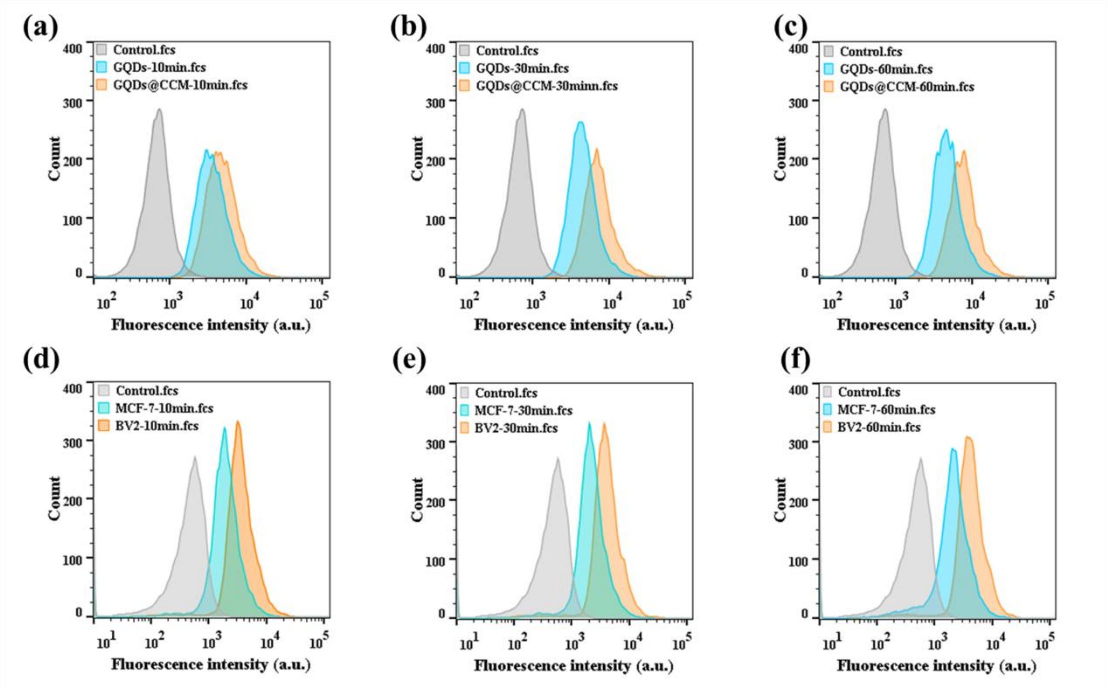

Figure S2. Flow cytometry analysis. (a, b and c) Mean fluorescence intensity of BV2 cells after 10, 30, and 60 min incubation with GQDs or GQDs@CCM, the final concentration of GQDs were 200 μg/mL. (d, e and f) Mean fluorescence intensity of MCF-7 and BV2 cells after 10, 30, and 60 min incubation with GQDs@CCM, the final concentration of GQDs were 200 μg/mL.

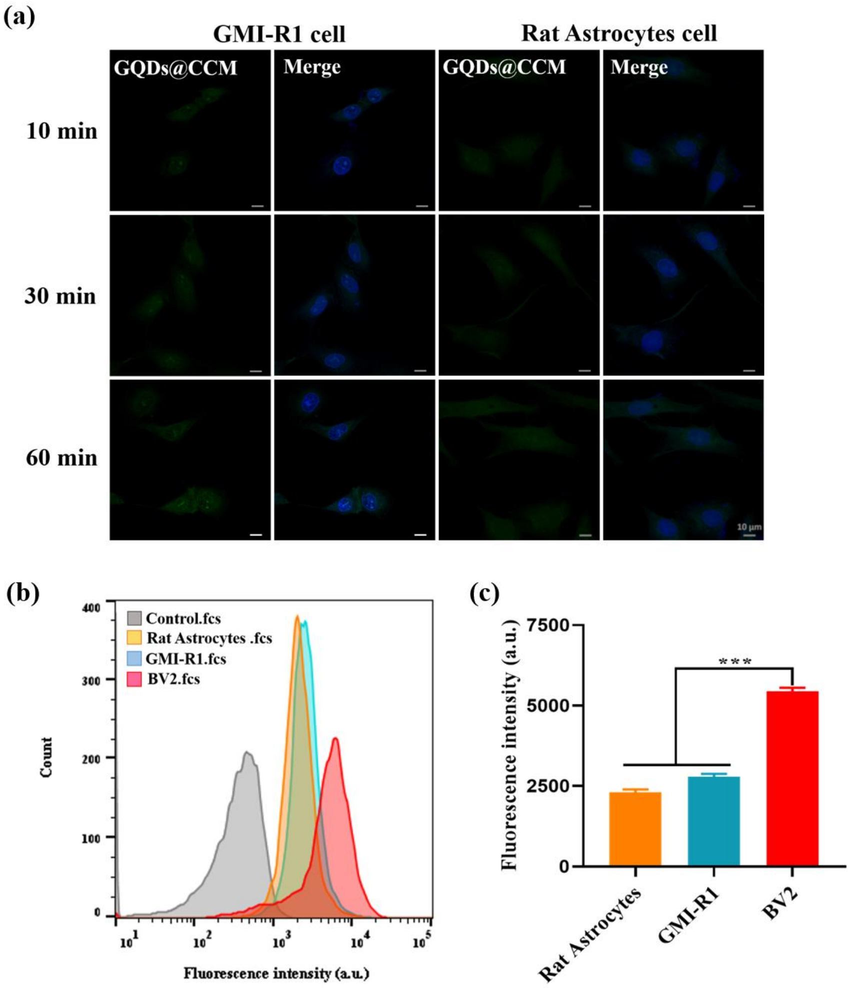

Figure S3. Homologous targeting of GQDs@CCM. (a) CLSM images of GMI-R1 and Rat astrocytes cells incubated with GQDs@CCM at GQDs concentration of 200 μg/mL for different time period. Scale bar = 10 μm. (b,c) Quantitative analysis of cell uptake by FCM in different cell lines.
